# An unexpected bronchoscopic finding possibly induced by extracorporeal membrane oxygenation: A case report of bronchial Dieulafoy disease

**DOI:** 10.1097/MD.0000000000039636

**Published:** 2024-09-06

**Authors:** Xiaochun Lv, Caibao Hu, Qianghong Xu, Min Fang

**Affiliations:** a Intensive Care Unit, Zhejiang Hospital, Hangzhou, Zhejiang, China.

**Keywords:** abnormal submucosal artery, bronchial Dieulafoy disease, extracorporeal membrane oxygenation, respiratory tract bleeding

## Abstract

**Rationale:**

Bronchial Dieulafoy disease (BDD), a rarely reported disease, comes from dilated or abnormal arteries under the bronchial mucosa. Patients with BDD are generally asymptomatic so this disease is frequently misdiagnosed. However, the submucosal arteries may dilate and rupture for various reasons, leading to recurrent respiratory tract bleeding and potentially life-threatening conditions. With the change of reversible factors such as intravascular pressure, the arteries may return to normal, allowing patients to recover to an asymptomatic state. This phenomenon has not been mentioned and concerned in previous studies, but it may have important implications for our correct understanding of this disease.

**Patient concerns:**

A 44-year-old female was admitted to intensive care unit with recurrent malignant arrhythmias. With the assistance of VA-extracorporeal membrane oxygenation (ECMO), both her vital signs and internal environment were all gradually stabilized. However, she had been experiencing recurrent respiratory tract bleeding. While removing the bloody secretion with a fiber bronchoscopy, a congested protruding granule on the wall of the patient’s left principal bronchus was found.

**Diagnosis:**

The patient was diagnosed with BDD and the granule was thought to be an abnormal artery of BDD.

**Interventions:**

For the patient’s condition, we did not implement any targeted interventions with the abnormal artery.

**Outcomes:**

After the weaning of VA-ECMO, the patient’s granule could not be found and the bleeding had also disappeared. She gradually weaned off the mechanical ventilation and was transferred to the Department of Cardiology. Then the patient was discharged after her condition stabilized. In more than half a year, the patient is in a normal physical condition.

**Lessons:**

The appearance and disappearance of abnormal artery is an interesting phenomena of BDD. The change of intravascular pressure due to various causes such as VA-ECMO may be the primary factor of it.

## 1. Introduction

Dieulafoy disease, characterized by rupture and bleeding of an abnormal submucosal artery, typically affects the digestive tract.^[1]^ In 1898, the French physician Dieulafoy first reported this condition and termed it “exulceratio simplex,” believing it to be an early stage of a gastric ulcer that could progress to hemorrhage.^[2]^ Bronchial Dieulafoy disease (BDD) refers to dilated or abnormal arteries under the bronchial mucosa. Given that the abnormal artery travels through the bronchial wall and is covered with a thin layer of mucosal epithelium, it is prone to rupture and bleeding due to various causes.^[3]^ While most abnormal arteries originate from the bronchial arterial system, a few originate from the pulmonary arterial system.^[4]^ BDD was first reported by Sweerts in 1995, with only a limited number of cases reported to date.^[5]^ Patients with BDD are generally asymptomatic but may manifest as recurrent respiratory tract bleeding. Notably, BDD is frequently misdiagnosed.^[6]^ Thus, this article aimed to outline the case of a female patient who received extracorporeal membrane oxygenation (ECMO) for recurrent malignant arrhythmia and was diagnosed with BDD following pulmonary hemorrhage. Interestingly, the abnormal artery spontaneously resolved without targeted interventions after the weaning of ECMO, warranting further discussion and investigation.

## 2. Case presentation

**Figure 1.**
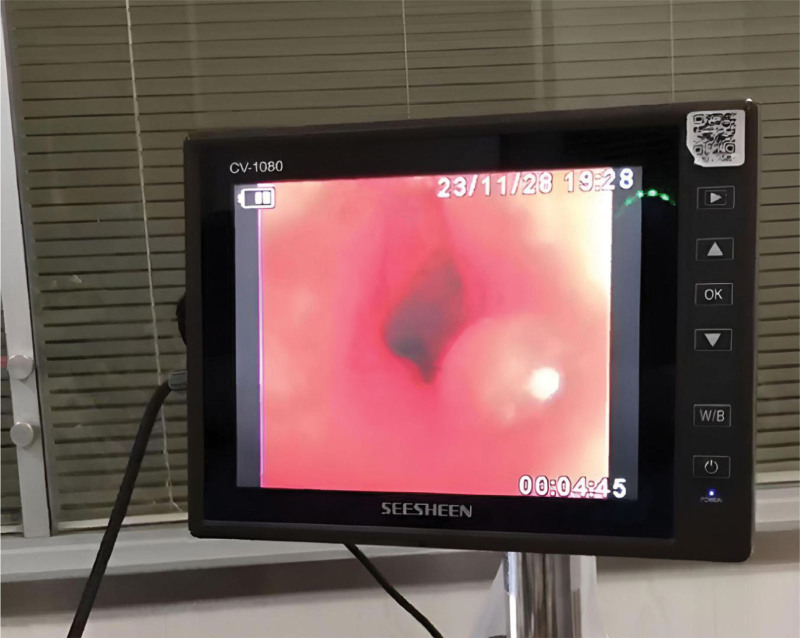
The patient’s congested protruding granule on the wall of her left principal bronchus.

**Figure 2.**
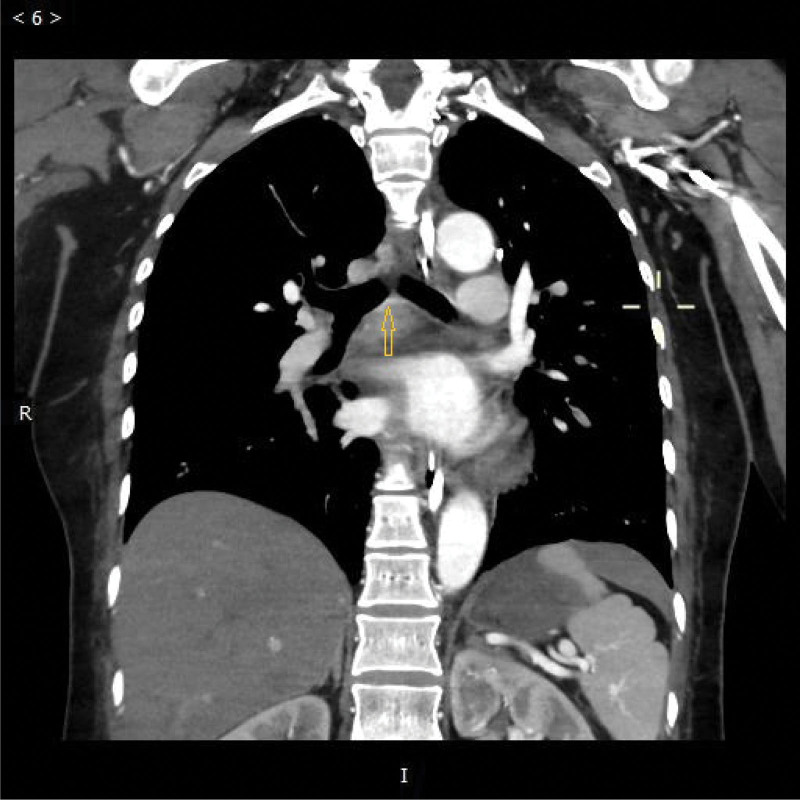
The patient’s abnormal artery around the left principal bronchus of the pulmonary vascular CTA (a, the yellow arrow points to this abnormal artery).

**Figure 3.**
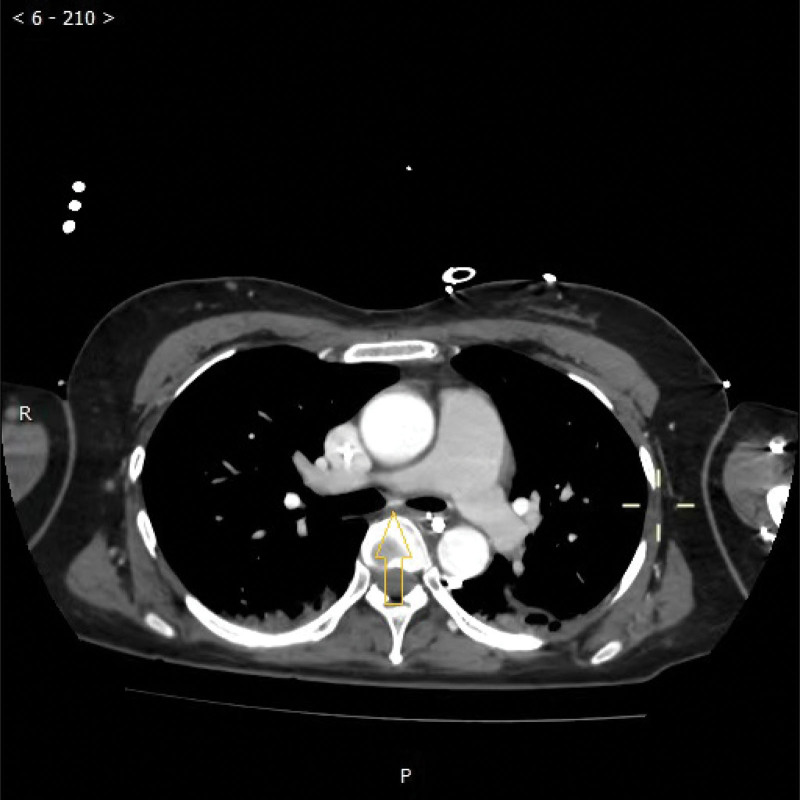
The patient’s abnormal artery around the left principal bronchus of the pulmonary vascular CTA (b, the yellow arrow points to this abnormal artery).

A 44-year-old female patient with no medical, family, or psychosocial history was admitted to our hospital for loss of consciousness. At 23:45, the patient suddenly experienced loss of consciousness at home without evidence of triggers. Her husband urgently contacted emergency services and performed cardiopulmonary resuscitation (CPR) as instructed. The ambulance arrived shortly thereafter, and the medical team took over CPR and transported the patient to our hospital. Upon arrival in the emergency room of our hospital, the patient experienced alternating pulseless ventricular tachycardia and ventricular fibrillation, as evidenced by the ECG monitor. Urgent tracheal intubation and electrical defibrillation were performed, and the patient was administered 1 mg epinephrine and 300 mg amiodarone to restore spontaneous circulation. At 00:29, the patient achieved return of spontaneous circulation (ROSC) and was transferred to the ICU for further treatment, where the patient was placed on mechanical ventilation and deep sedation. Coagulation function tests revealed a D-Dimer level of 6.40 mg/L, PT of 13.9 seconds, APTT of 37.0 seconds, TT of 17.2 seconds, FIB level of 2.32 g/L, INR of 1.09, PLT count of 227 × 10^9^/L, MPV of 10.6 fL, and PCT of 0.240 %, with no significant abnormalities. Meanwhile, arterial blood gas analysis (ABG) showed a pH value of 7.298, a BE of −5.6 mmol/L, an HCO^3−^ of 20.7 mmol/L, and a lactic acid level of 10.48 mmol/L. At the same time, cardiac ultrasound displayed an LVEF of approximately 30% to 40%. Then, the patient was administered mild hypothermia therapy, anti-infective agents, anti-inflammatory drugs, PPIs, and neuroprotective drugs. Despite these interventions, the patient experienced recurrent episodes of pulseless ventricular tachycardia and ventricular fibrillation, with normal blood potassium levels. Given the severity of the electrical storm, an electrophysiological examination was considered. However, potential challenges of this approach included the risk of inducing malignant arrhythmias during this procedure and identifying the ablation target for ventricular fibrillation. Despite the successful restoration of spontaneous circulation via repeated electrical defibrillation and CPR, ECMO was considered to stabilize her vital signs.^[7]^ Therefore, VA-ECMO was initiated, with a blood flow rate of about 2.5 to 3.0 L/min and a fresh oxygen flow rate of about 5 L/min. With the assistance of VA-ECMO, both her vital signs and internal environment were gradually stabilized, and other therapies were maintained according to the original protocol. However, at the same time, significant bloody secretions were observed in the respiratory tract. Fiberoptic bronchoscopy illustrated that these secretions could be found in the main bronchi on both sides and extended to the lobar, segmental, and subsegmental bronchi. On the 6th day of hospitalization, a congested, protruding granule with a diameter of roughly 8 mm was detected on the wall of the left principal bronchus (Fig. 1). Moreover, pulmonary artery CTA showed the presence of transverse blood vessels beneath the mucosa of the left main bronchus (Figs. 2 and 3). In addition, we did not see any obvious injury of the sternum or ribs on CT, so we did not believe that the bleeding was caused by CPR. To confirm the diagnosis and rule out bleeding due to ANCA-associated vasculitis, the patient was tested for ANCA-associated antibodies. As anticipated, the results were negative for c-ANCA, p-ANCA, x-ANCA, PR3 Ab-IgG, and MPO.

A respiratory specialist in our hospital was consulted, and a preliminary diagnosis of BDD was made. Based on her clinical manifestations and imaging evidence, we obtained the diagnosis. Due to the unstable circulatory status of the patient and the absence of severe anemia or coagulation disorders, we only adopted symptomatic treatments such as correcting coagulation function and local airway hemostasis, without selective vascular embolization. As the patient’s condition progressively improved, she was weaned off ECMO. Nevertheless, fiberoptic bronchoscopy examination conducted the following day did not detect any granule, and the bleeding almost disappeared. Thus, mechanical ventilation was weaned, and the endotracheal tube was removed within the next few days. Finally, the patient was successfully transferred to the Department of Cardiology for further assessment. There, she underwent a coronary CTA to investigate the cause of malignant arrhythmia, but no evidence of abnormalities was found. Subsequently, the doctor recommended the implantation of cardioverter defibrillator. However, the patient refused and was discharged after her condition stabilized. During the follow-up period, the patient did not experience airway bleeding again.

## 3. Discussion

At present, both the etiology and pathogenesis of BDD remain elusive. It may be related to congenital abnormalities of the broncho-pulmonary arteries, chronic airway inflammation, or injury.^[4]^ Notably, earlier studies described that BDD is linked to long-term smoking.^[8]^ However, other studies established that a large proportion of patients with BDD have no history of smoking or primary respiratory disease.^[9]^ The clinical characteristics of BDD are as follows: (1) It is commonly associated with massive hemoptysis of unknown origin or with nodular lesions in the lumen of the bronchus identified during fiberoptic bronchoscopy. Of note, these lesions may cause massive bleeding after biopsy.^[10]^ (2) It is prevalent in adult males, although cases have also been reported in adolescents and children. The male-to-female ratio is about 2:1.^[9,11–13]^ (3) It is more commonly identified in the right lung, with bilateral involvement being rare.^[12]^ (4) The volume of blood loss significantly varies between cases, ranging from 20 mL to approximately 2000 mL.^[12,14]^ (5) The lesions are mostly focal and solitary.^[15]^ (6) Routine blood tests and coagulation profiles of patients with BDD are mostly normal, and the detection rate in chest imaging is generally low.^[11]^ (7) The diagnosis of BDD relies on pathological examination, which principally displays dilated or malformed arteries passing through the bronchial wall and adjacent to the bronchial lumen, covered with only the mucosal epithelium.^[13]^ Resection of the focal pulmonary lobe may be a radical treatment for BDD. However, for most patients who are not eligible for such interventions, selective bronchial artery embolization is another viable approach. Nevertheless, embolization is associated with recurrence and failure.^[6]^ Barisione et al reported that only 50% of patients undergoing selective artery embolization achieved complete recovery.^[16]^ It may be ascribed to the abnormal arteries of some patients originating from the pulmonary artery.^[17]^

Noteworthily, several challenges were encountered in the diagnosis of the patient. ANCA-associated vasculitis was initially considered.^[18,19]^ However, the patient presented with massive endobronchial hemorrhage instead of diffuse alveolar hemorrhage, and antibody test results were all negative. Subsequently, biopsy was considered, but past experience indicated that biopsy may lead to excessive bleeding,^[10]^ which could be fatal for a patient experiencing hemoptysis. Unexpectedly, the final fiberoptic bronchoscopy did not display evidence of the previously detected protruding granule under the left bronchial mucosa despite the absence of specific interventions such as vascular embolization, clamping, or laser treatment. We also considered the possibility that the vessel was a submucosal artery with a narrow internal diameter and normal blood flow. Under physiological conditions, it did not exhibit tortuous expansion or mucosal bulging and remained undetectable. Following the initiation of VA-ECMO, the advective blood flow from the ECMO catheter opposed the natural cardiac ejection flow, resulting in high intravascular pressure. As the cardiac activity of the patient improved, resistance to the advective blood flow from VA-ECMO similarly increased, and the increased pressure may have induced the bronchial artery to dilate in a tumor-like morphology. After the discontinuation of VA-ECMO and the normalization of hemodynamics, the pressure within the dilated submucosal artery decreased, thereby allowing the submucosal artery to return to its original state.^[20–22]^ This may have accounted for the disappearance of the dilated artery after weaning off ECMO. Indeed, it had not disappeared but reverted to its baseline state owing to the decrease in intravascular pressure.

## 4. Conclusions

BDD is a rare disease that causes severe and potentially fatal bleeding in the respiratory tract. Its diagnosis and treatment all have inherent limitations. Due to the rarity of BDD, related studies are scarce. This case report outlines an interesting phenomenon where the abnormal artery disappeared spontaneously during treatment. This phenomenon could be ascribed to fluctuations in intravascular pressure induced by various factors such as the influence of VA-ECMO. This viewpoint provides a reference for us to treat and analyze similar patients. Of course, there are some limitations in this case report. Firstly, the patient’s coronary CTA showed a negative result, and no electrophysiological examination was performed, making it impossible to determine the cause of recurrent malignant arrhythmias. Secondly, the bronchial submucosal artery have returned to its original state, so we do not know whether the artery will undergo tortuous dilation again in the future and cause respiratory bleeding. If the patient accepted the selective arterial embolization actively at that time, the probability of subsequent bleeding would be greatly reduced.

## Author contributions

**Conceptualization:** Xiaochun Lv.

**Formal analysis:** Xiaochun Lv.

**Supervision:** Qianghong Xu.

**Writing – original draft:** Xiaochun Lv, Min Fang.

**Writing – review & editing:** Caibao Hu.
